# The Effects of Storm Runoff on Water Quality and the Coping Strategy of a Deep Canyon-Shaped Source Water Reservoir in China

**DOI:** 10.3390/ijerph120707839

**Published:** 2015-07-10

**Authors:** Weixing Ma, Tinglin Huang, Xuan Li, Zizhen Zhou, Yang Li, Kang Zeng

**Affiliations:** School of Environmental and Municipal Engineering, Xi’an University of Architecture and Technology, Xi’an 710043, China; E-Mails: hs_weixing@163.com (W.M.); lixuan8781687626@sohu.com (X.L.); zhouzizhen001@sina.com (Z.Z.); liyang1989526@163.com (Y.L.); zengkangxt@163.com (K.Z.)

**Keywords:** rainstorm, reservoir, water quality, water-lifting aerator, mixing

## Abstract

Storm runoff events in the flooding season affect the water quality of reservoirs and increase risks to the water supply, but coping strategies have seldom been reported. The phenomenon of turbid current intrusion resulting in water turbidity and anoxic conditions reappearing after storm runoff, resulting in the deterioration of water quality, was observed in the flooding season in the deep canyon-shaped Heihe Reservoir. The objective of this work was to elucidate the effects of storm runoff on the Heihe Reservoir water quality and find a coping strategy. In this study, an intensive sampling campaign measuring water temperature, dissolved oxygen, turbidity, nutrients, and metals were conducted in the reservoir over a period of two years, and the water-lifting aerators were improved to achieve single aeration and a full layer of mixing and oxygenation functions using different volumes of gas. The operation of the improved water-lifting aerators mixed the reservoir three months ahead of the natural mixing time, and good water quality was maintained during the induced mixing period, thereby extending the good water quality period. The results can provide an effective coping strategy to improve the water quality of a source water reservoir and ensure the safety of drinking water.

## 1. Introduction

A source water reservoir is different from a general lake reservoir in that its requirements for water quality are higher; therefore, it is critical to improve the water quality and control the pollution of source water reservoirs [1,2].

It is predicted that the frequency of extreme rainfall events will occur more frequently than the mean precipitation rate in the near future [3,4], while the amount of total precipitation is predicted to change only slowly [5]. Problems of high outflow turbidity and sediment deposition in reservoirs often accompany storm runoff [6,7]. A significant density of turbid flow follows high rainfall events in many lakes and reservoirs [8]. The inflowing water will enter the reservoir as a plunging underflow if the density of the inflow water is significantly higher than that of the reservoir, and it may flow into the bottom and fill up the reservoir from the bottom if the inflow density is large enough [9,10].

Significant turbid currents coupled with a large amount of dissolved oxygen (DO) entering the bottom of the reservoir can effectively inhibit the release of contaminants from the sediments [11]. However, the input of easily degraded organic matter can lead to an increase in the oxygen consumption rate in the water, causing an increased release of pollutants from the sediment once the hypolimnion becomes anoxic again [12]. Reasonably releasing density flow and selecting the appropriate intake height would be effective solutions to the high turbidity problems during the flood season [13,14].

Hypolimnetic oxygen depletion after a rainstorm can result in the release of soluble chemical species from the sediments, deteriorating water quality, and increasing drinking water treatment costs [15,16]. The lack of oxygen at the bottom is a key factor of endogenous pollution in the reservoir, so improving the reservoir bottom dissolved oxygen content can effectively inhibit the release of pollutants from the sediments [17]. There are two ways to improve the concentration of dissolved oxygen at the bottom. One way is to destroy the thermal stratification structure, so that the surface water with high dissolved oxygen can be transported to the lower layer, increasing the dissolved oxygen in this water [18,19,20,21]. The other is hypolimnetic oxygenation, which can directly oxygenate the bottom hypoxic water without destroying the stratification structure [15,22,23]. The water-lifting aerator was developed to oxygenate the bottom water and mix the water in the upper and lower layers in stratified reservoirs where pollutants increase from sediments because of anoxic conditions in the bottom water [24,25,26,27].

The Heihe Reservoir is the most important raw water source for Xi’an City, accounting for 76% of the total water supply to this city. Eight water-lifting aerators (WLAs) have been successfully applied in this reservoir to restrain algal growth, increase the dissolved oxygen in the lower water layer, and inhibit the release of pollutants from the bottom sediments, thus ensuring the water quality during the summer stratified period. In recent years, significant turbid density flows followed large rainfall events, leading to the deterioration of reservoir water quality and influencing the operation of WLAs during the flooding season. The water managers of the Heihe Reservoir suspend operation of the WLAs during storm runoff periods to avoid increasing the turbidity of the upper layers and select an appropriate intake height to avoid high outflow turbidity. However, anoxic conditions reappeared after the storm runoff ended, caused by the increase of the oxygen consumption rate that could accelerate the release of pollutants from the bottom sediment. The objective of this work was to gain a fuller understanding of the effects of storm runoff on the Heihe Reservoir water quality and to find a coping strategy that can address the lack of oxygen at the bottom after storm rainfall events. To address this issue, an intensive sampling campaign measuring water temperature, dissolved oxygen, turbidity, nutrients, and metals was conducted at the reservoir over a period of two years, and the improved WLAs were operated. The results can provide theoretical guidance and technical support for improving reservoir water quality and ensuring outflow water quality during a storm runoff period.

## 2. Materials and Methods

### 2.1. Study Site

**Figure 1:**
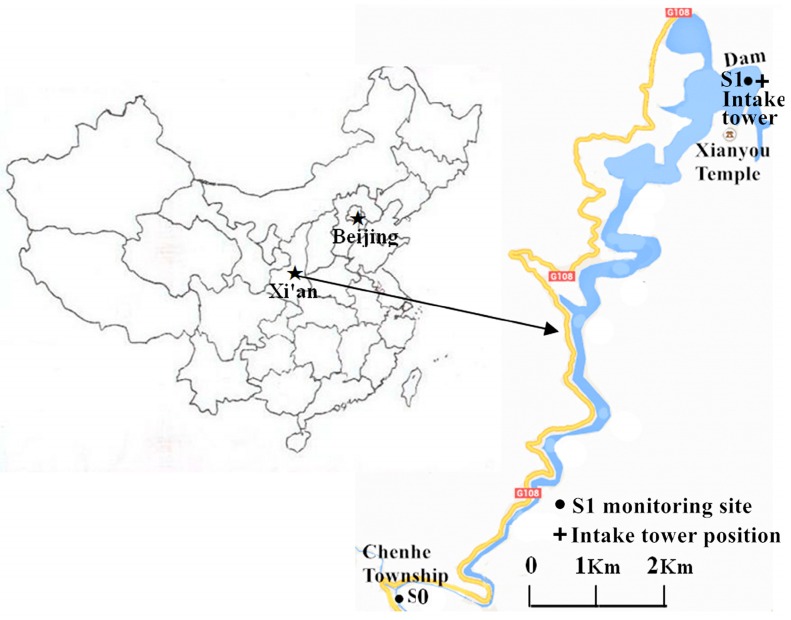
Location of the Heihe Reservoir and the sampling sites.

The Heihe Reservoir (34°42′–34°13′ N; 107°43′–108°24′ E) is a large canyon-shaped reservoir located in Zhouzhi County, approximately 86 km southwest of Xi’an, in Shanxi Province, in the northwest of China (Figure 1). Xi’an City is the capital of Shaanxi province, located in the southern part of the Guanzhong Plain, with a permanent resident population of 8.63 million (2014). With the Qinling Mountains to the south and the Weihe River to the north, it has a semi-moist monsoon climate with a clear distinction between the four seasons. The Heihe Reservoir is the most important raw water source for Xi’an City, with a daily water supply of 8.0 × 10^5^ m^3^. When full, this reservoir has a total capacity of 2.0 × 10^8^ m^3^, with a surface area of 4.55 km^2^. The reservoir is very deep, with mean and maximum depths of 44 m and 94 m, respectively. Generally, the normal high water level is 594 m above sea level, and the lowest allowed water level is 520 m above sea level.

Originating from the Qinling Mountain, the Heihe River is the main stream of the Heihe Reservoir, draining a catchment area of 1481 km^2^. The upstream and surrounding landscapes of the reservoir are largely unmodified and over 70% of them consist of forests and mountains. The annual average rainfall is above 900 mm, and over 60% of the annual precipitation is centralized in the flooding season (July to September). In recent years, the water quality problem aroused by rainstorms has become severe, including landslides and debris flow, which cause an increasing content of suspended solids, leading to turbidity intrusion in the flooding season and high turbidity in the Heihe Reservoir, presenting an acute water quality problem to Xi’an City. According to the Chinese national drinking water standard, a large amount of coagulant has been added to the treated water, which not only increases water treatment costs for water plants but also seriously affects the city’s water supply safety.

### 2.2. Field Observation

**Figure 2:**
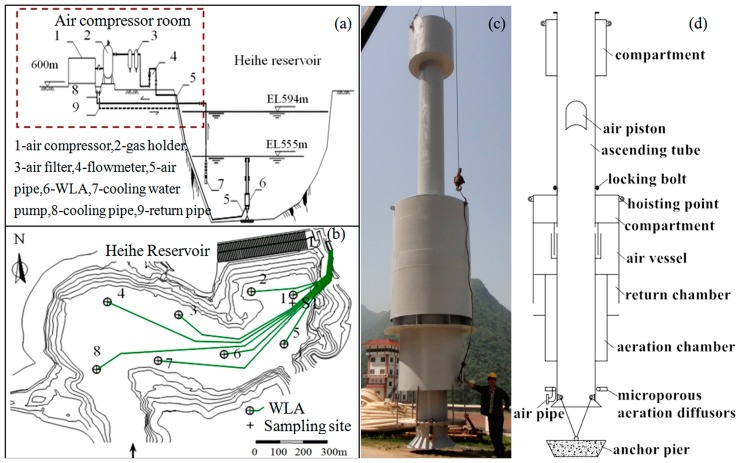
The water-lifting and aeration system: Flow chart of the water-lifting and aeration system (**a**); layout of the water-lifting aerators in Heihe Reservoir (**b**); the photo of the assembled water-lifting aerator (**c**) and the diagram of the water-lifting aerator structure (**d**).

Weekly sampling campaigns were conducted from 2012 to 2014 to investigate the water quality changing process at the main reservoir near the dam (S1 monitoring site), and more frequent investigations took place during the flooding season. Three monitoring sites were chosen for quality monitoring during the improved WLAs operation period (Figure 2). Every week, vertical profiles of water temperature, dissolved oxygen, water turbidity, and chlorophyll-a were measured using Hydro-lab DS5 (Hach, USA), which was calibrated every three months. Samples from the monitoring sites were taken at 5 m intervals from the surface (0.5 m under the water surface) to the bottom (0.5 m above the sediment). All samples were stored at 4 °C immediately after collection. The total nitrogen (TN), total phosphorus (TP), total iron (Fe) and manganese (Mn) were measured within 48 h using officially recommended methods of analysis [28].

Daily hydrological and climate data, *i.e.*, reservoir water levels, water inflow, water discharge and watershed precipitation during the investigation period were obtained from measurements by the Heihe Reservoir hydrological station adjacent to the dam.

### 2.3. The Improvement of the Water-Lifting Aerators in the Heihe Reservoir

The water-lifting aerators were installed in the Heihe reservoir in 2010. The flow chart and layout of the WLAs system in Heihe reservoir are shown in Figure 2a,b. The compressed air produced by the air compressor is transported to the gas holder, which is a regulator system that maintains the compressed air pressure. Then, the compressed air flows through the air filter, where the oil contamination and other impurities are separated. After the air filter, the main air supply pipe is divided into eight separate pipes. The valve controls the air flow, and the flowmeter displays the air flow, as the compressed air is transported to each water-lifting aerator via the air pipe. Using the water-cooled compressor, the cooling water is transported to the air compressor via the cooling water pump in the reservoir, and then the cooled water returns to the reservoir.

The mixing function of the WLAs can restrain algal growth, break up water stratification, and increase the concentration of DO in the bottom water. However, the mixing of the upper and lower water layers during a storm runoff period could lead to high turbidity of the upper water and increased outflow turbidity. To solve this problem, the structure of the air vessel was improved to achieve single aeration at the bottom using a small volume of gas (<20 m^3^/h) and achieving a full layer’s mixing and oxygenation functions using a large volume of gas. The perforated aeration tube was replaced with a microporous aeration device to strengthen the oxygenation to the hypolimnion. The microporous aeration device releases compressed air as tiny bubbles, enlarging the contact area between the air and water in the aeration process, significantly improving the oxygen transfer efficiency [29].

A photo of the improved water-lifting aerator and a diagram of the water-lifting aerator structure are shown in Figure 2c,d, respectively. Each WLA is fixed to an anchor pier at the reservoir bed. The compressed air is continuously delivered to the microporous aeration diffusor and then released to the aeration chamber in the form of small bubbles (with a diameter of less than 10 μm). The aeration chamber and return chamber provide the bubbles with sufficient time to deliver the oxygen to the water. After oxygenation, the bubbles advance the water to the bottom of the reservoir via the return chamber. This function can directly oxygenate the water in the lower layer and circulate the lower, anoxic water to promote the diffusion of dissolved oxygen. The off-gas was collected in the air vessel. When the air vessel is full, the gas is instantaneously released to the ascending tube. Subsequently, a large air piston forms, rises promptly, and then accelerates the water in the ascending tube and part of the water in the lower layer, which continues to rise until the air piston exits the outlet of the ascending tube.

In *in-situ* experiments, we added 35 holes, each with a diameter of 2 mm, to the outside of the air vessel, which can ensure that the large air piston does not form under a small volume of gas (<20 m^3^/h).

## 3. Results and Discussion

### 3.1. Climate and Hydrological Conditions

**Figure 3:**
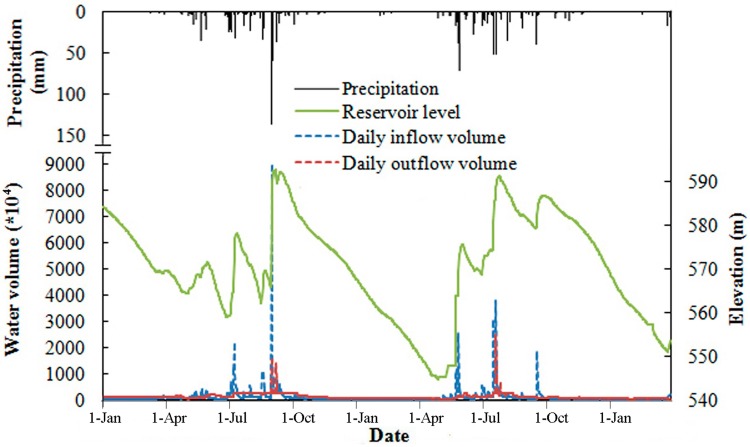
Variations in daily precipitation, inflow volume, outflow volume, and water level in the Heihe Reservoir from 1 January 2012 to 31 March 2014.

Figure 3 shows the daily precipitation, water level, inflow and outflow volume from 1 January 2012 to 31 March 2014. The annual total precipitation was 812.42 mm in 2012 and 738.5 mm in 2013, with more than 60% of the annual precipitation recorded between July and September (flood season). In general, the precipitation and inflow volume are correlated, especially during the period of the flood season, when enough rain falls to saturate the catchment. As shown in Figure 3, an extremely heavy rainfall event occurred from 31 August to 2 September in 2012, with a total rainfall of 219 mm. The total water inflow of this rainfall event was 115 million m^3^, with the largest peak flow reaching 1750 m^3^/s, the largest flood peak since 2005. Compared to 2012, the rainstorms in 2013 were most severe in July, with the daily inflow volume reaching 30.17 million m^3^ and 37.83 million m^3^ on 19 July and 23 July, respectively. 

The outflow supply for city water usually has a relativity small change over the year. During the flood period, the outflow volume increased mainly due to the need for flood control and power generation. The water level of the reservoir depends on the difference between the inflow volume and outflow volume; the seasonal reservoir water level variation was very great, ranging from EL 557.8 m to EL 594.08 m over the two-year period. Before the flood season, the water level of the Heihe reservoir had dropped to the lower water level. During the flood season, the reservoir water level can reach the normal high level; the water level is maintained at a high level from August to November because of the frequent rainfall. After the flood season, the water level declines until May of the next year.

### 3.2. Seasonal Variations of Temperature and Dissolved Oxygen

**Figure 4:**
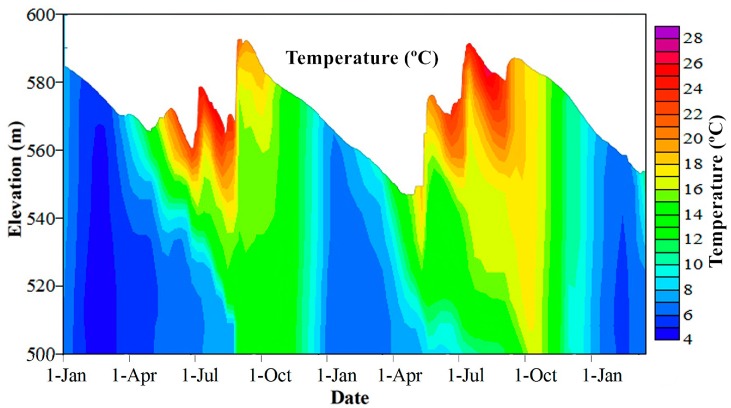
Vertical variation in temperature in the Heihe Reservoir from 1 January 2012 to 31 March 2014.

Temperature stratification in the Heihe Reservoir began in April and became stable in late May, with a rapid increase of the surface water temperature and a slow change in the bottom water temperature (Figure 4). It was strongest in August, with a maximum water temperature difference of over 20 °C. In general, the Heihe Reservoir was stratified for most of the year, with a short mixing period in the winter, when the surface water temperature fell below the bottom water temperature in mid-January. However, storm runoff can affect the stability of the temperature stratification, thus influencing the length of the stratified period.

**Figure 5:**
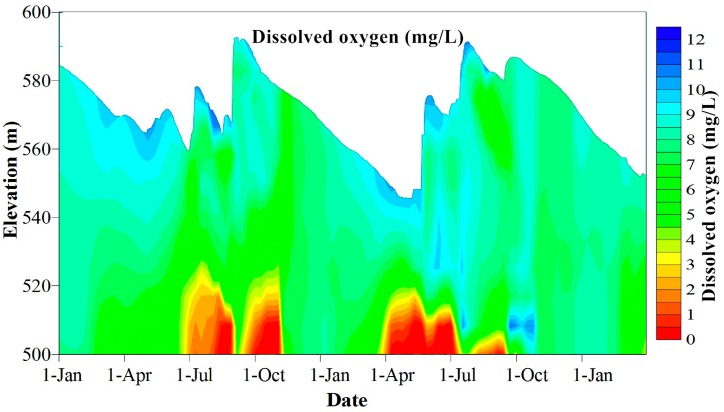
Vertical variation in dissolved oxygen in the Heihe Reservoir from 1 January 2012 to 31 March 2014.

The existence of a temperature gradient effectively restricts the heat and mass transfer processes among the vertical water layers. The available dissolved oxygen in the hypolimnion was rapidly reduced under the consumption of water and sediment and the hypolimnion became anoxic during the stratified period. In general, the hypolimnion water in the Heihe Reservoir became anaerobic in June and maintained anaerobic conditions until mid-January, when the reservoir water was completely mixed (Figure 5). Longer periods of anoxia resulted in a more significant deterioration of water quality as pollutants were released from the sediments.

### 3.3. Effects of Storm Runoff on Water Quality

#### 3.3.1. Effects of Storm Runoff in 2012

**Figure 6:**
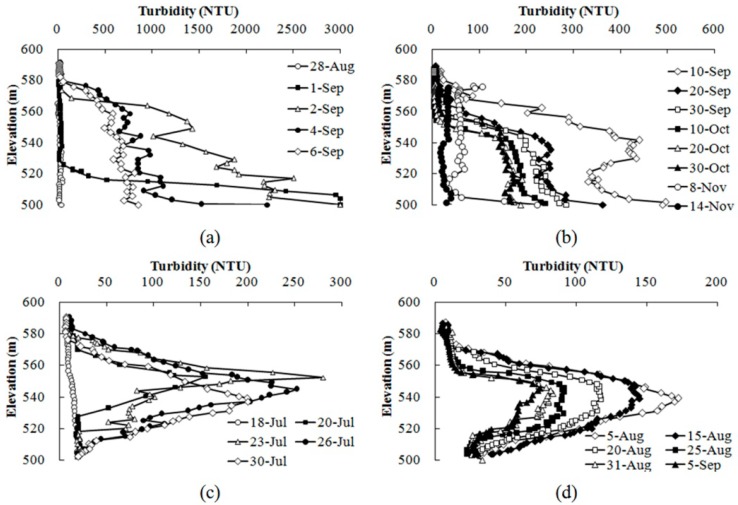
Vertical variation in turbidity during the flooding season in 2012 (**a**,**b**) and 2013 (**c**,**d**).

The terrain upstream of the Heihe Reservoir consists primarily of hills covered with forest with little human activity. The water quality in Heihe Reservoir can meet the requirement of the Chinese national surface water quality standard most of the time, with low nutrient and metal levels. As Figure 6a shows, the vertical turbidity of the Heihe Reservoir was lower than 10 NTU before the heavy rainfall event that occurred from 31 August to 2 September 2012.

Storm runoff has significant effects on water quality. As shown in Figure 4 and Figure 5, the storm runoff at the end of August in 2012 entered the reservoir as a plunging underflow, leading the bottom water temperature to increase from 7.3 °C to 15.2 °C and the dissolved oxygen in the bottom water increased from 0 mg/L to 9.02 mg/L. The high inflow of this storm runoff transported a large amount of suspended matter, leading to increased turbidity and density at the bottom, the density of the inflow water was larger than the background water, so the inflow will occur as underflow, and the turbidity of the underflow water was greater than 3000 NTU (the upper limit of the DS5 device).

The larger particles began to settle rapidly after the storm runoff, and water turbidity dropped from 3000 NTU to lower than 1000 NTU within a week (Figure 6a). The large amount of easily degraded organic matter transported into the reservoir by the storm runoff led to an increase in the oxygen consumption rate in the water, causing the water at the bottom of the reservoir to become anoxic again within 20 days of the storm runoff (Figure 5). Thereafter, the bottom water maintained its anaerobic state, until the reservoir mixed at the beginning of November due to the increased bottom water temperature.

The slow settling velocity of fine sediment caused the water turbidity in the hypolimnion (from EL 550 m to the bottom) to be higher than 100 NTU until natural mixing occurred in early November. The strong mixing effect led to an increase in the turbidity of the upper water, leading to the increase of outflow turbidity (Figure 6b).

**Figure 7:**
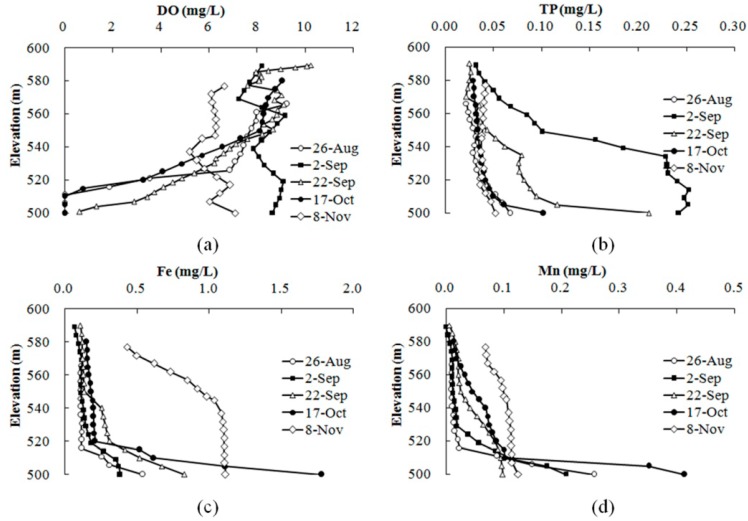
Vertical variation in DO (**a**), TP (**b**), Fe (**c**) and Mn (**d**) in the Heihe Reservoir at S1 in 2012.

The hypolimnion water became anoxic in July 2012 (Figure 5). Long period of anoxic conditions caused the concentrations of TP, Fe and Mn in the bottom water reached 0.067 mg/L, 0.54 mg/L and 0.26 mg/L, which was 0.34, 0.8, 1.6 times exceed the Chinese national surface water quality standard Class III, respectively, before the storm runoff at the end of August 2012 (Figure 7). The increased bottom DO caused by storm runoff effectively inhibited the further release of pollutants (2-Sep). Furthermore, the storm runoff transported large amounts of particulate nutrients into the reservoir, leading the TP in the bottom water to reach 0.242 mg/L during the storm runoff, which was 3.84 times corresponding to the Chinese national surface water quality standard Class III. The particulate TP easily settled in the reservoir, leading to an increased endogenous nutrient load. However, anoxic conditions reappeared after the storm runoff ended and accelerated the release of pollutants. The concentrations of TP, Fe and Mn at the bottom reached 0.102 mg/L, 1.78 mg/L and 0.41 mg/L, which was 1.04, 4.93, 3.1 times exceeding the Chinese national surface water quality standard Class III, respectively, on 17 October. The mixing of the whole reservoir in November led to increased concentrations of pollutants in the upper water.

#### 3.3.2. Effects of Storm Runoff in 2013

In 2013, the storm runoff in July entered the reservoir as interflows. The vertical disturbance was intensified by the high inflow volumes, thus increasing the heat and mass transfer processes in the hypolimnion and leading the bottom water temperature to increase from 10.3 °C to 11.2 °C (Figure 4), the dissolved oxygen increased from 0 mg/L to 6.3 mg/L (Figure 5). The largest peak flow was 835 m^3^/s in July and the inflow density was larger than the surface background water, but lighter than the bottom background water, so the inflow will occurred as interflow, and the position of the undercurrent was approximately EL 550 m. The water turbidity slowly dropped from 280 NTU to less than 100 NTU within a month (Figure 6c,d).

The hypolimnion water became anaerobic in April due to the influx of suspended matter in 2012. Similar to the case of 2012, as shown in Table 1, deterioration of water quality at the bottom was monitored over the long period of anoxic conditions, and the concentrations of TP and Fe reached 0.085 mg/L and 0.63 mg/L, which was 0.7 and 1.1 times exceeding the Chinese national surface water quality standard Class III, respectively, on 10 July. Storm runoff in July led the dissolved oxygen in the hypolimnion to increase, which effectively inhibited the further release of endogenous pollution. However, high inflow volumes carrying large amounts of solids and organic matter led to the TP and Fe concentrations to increase. The concentrations of TP and Fe in the undercurrent layer (elevation of 540 m) reached 0.065 mg/L and 0.67 mg/L, respectively.

The high loads of solids and organic matter put into the reservoir by the storm runoff and the increase of water temperature in the hypolimnion after the storm runoff increased the water column and sediment oxygen consumption rate. The bottom water became anoxic again after the storm runoff on 12 September, which was subsequently followed by a sharp increase in nutrient and metal concentrations. Similar to the situation in 2012, the strong convective mixing effect would transport hypolimnion water with high concentrations of pollutants to the upper water, leading to the deterioration of the whole reservoir water quality during the overturn period. It can be predicted that the deterioration of water quality would be severe after 12 September.

Due to the difference in rainfall between years, the total inflow volume was different and it was 0.93 billion m^3^ in 2011, 0.54 billion m^3^ in 2012, and the water exchange period was about 2.6 months in 2011 and 4.4 months in 2012.

**Table 1:** Vertical distribution of temperature, DO, TP and Fe in the Heihe Reservoir at S1 in 2013.

Elevation	T (°C)			DO (mg/L)			TP (mg/L)			Fe (mg/L)		
(m)	10-July	23-July	12-September	10-July	23-July	12-September	10-July	23-July	12-September	10-July	23-July	12-September
588.7	/	23.43	/	/	10.42	/	/	0.014	/	/	0.13	/
580	22.30	19.21	23.59	9.5	8.02	8.57	0.013	0.012	0.010	0.09	0.18	0.03
570	21.73	16.97	22.59	9.19	8.60	7.42	0.011	0.021	0.011	0.10	0.30	0.03
560	20.96	16.42	20.24	8.78	9.00	5.73	0.018	0.043	0.010	0.12	0.45	0.03
550	17.91	15.86	17.28	7.89	9.05	7.70	0.015	0.045	0.011	0.15	0.54	0.07
540	15.14	15.22	16.53	8.62	8.84	7.73	0.022	0.065	0.053	0.12	0.67	0.67
530	13.71	14.86	16.21	8.63	8.71	7.60	0.023	0.050	0.038	0.19	0.44	0.49
520	13.18	14.32	16.00	9.04	8.55	7.15	0.031	0.048	0.031	0.23	0.46	0.43
510	12.49	13.08	13.16	7.85	8.82	5.31	0.067	0.048	0.045	0.48	0.41	0.44
500	10.32	11.24	11.67	0.00	6.31	0.10	0.085	0.046	0.055	0.63	0.48	0.43

10/23-July represent before/after the rainstorm, 12-September represent when the hypolimnion became anaerobic again. The elevations of 10-July, 23-July, and 12-September were EL 573 m, EL 588.7 m, and EL 580 m, respectively.

### 3.4. Water Quality Improvement during the WLAs Operation Period

The improvement of the water-lifting aerator system began in early May 2013 and was completed at early July. The commissioning of the improved water-lifting aerator occurred from 2 July to 19 July. During the period of commissioning, the volume of compressed air was increased gradually from 10 m^3^/h to 40 m^3^/h. The improved water-lifting aerator achieved single aeration with a small volume of gas (<20 m^3^/h) and achieving the full layer’s mixing and oxygenation with a large volume of gas. All eight water-lifting aerators were running well and were stable during the commissioning period, which was in accordance with our preset goal.

**Figure 8:**
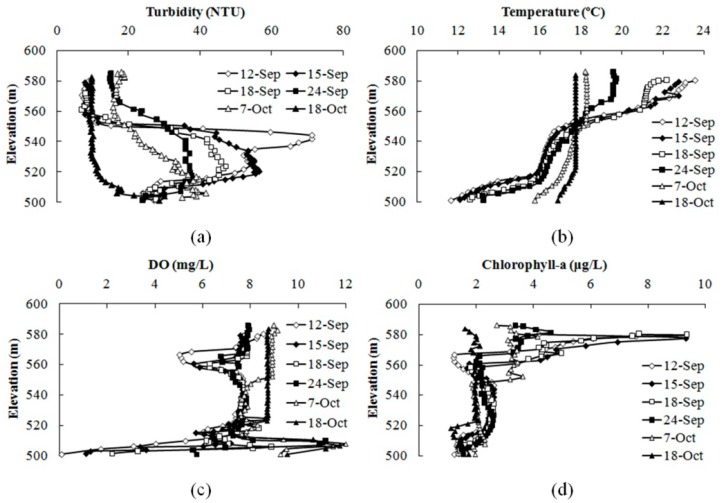
Vertical variation in turbidity (**a**), temperature (**b**), DO (**c**), and chlorophyll-a (**d**) in the Heihe Reservoir during the WLAs operation period.

Influenced by the storm runoff occurring on 19 July and 23 July in 2013, the dissolved oxygen in the bottom water increased from 0 mg/L to 6.3 mg/L. Thus, the favorable conditions induced by the storm runoff were exploited by suspending operation of the WLAs to reduce power consumption. On 12 September, the dissolved oxygen in the bottom water decreased to 0.1 mg/L. Then, the improved water-lifting aerator began operation on 13 September to inhibit the release of pollutants from the bottom sediments. According to the vertical distribution of the water turbidity, the WLAs first ran at a small air flow rate to avoid causing an increase of the turbidity in the upper water. When the turbidity fell to below 50 NTU on 18 September, then the equipment was operated with an air flow of 35–40 m^3^/h to restrain algal growth and accelerate the destratification processes. The vertical distributions of turbidity, temperature, DO, and chlorophyll-a during the WLAs operation period are shown in Figure 8.

#### 3.4.1. Single Aeration in the Hypolimnion

The water-lifting aerators operated with an air flow rate of approximately 15–18 m^3^/h to achieve single aeration in the hypolimnion from 13 September to 18 September. As Figure 8a shows, the turbidity in the hypolimnion dropped from 72 NTU to 46 NTU during this period, indicating that the operation of WLAs would not disturb the sediments at the bottom. Without the mixing of the surface water with the hypolimnion water, the turbidity in the upper water was lower than 10 NTU (Figure 8a). The turbulence in the hypolimnion led to a slight increase in the bottom water temperature but was not substantial enough to significantly disrupt the thermal stratification (Figure 8b). The microporous aeration device improved the dissolved oxygen transfer efficiency, leading to a high concentration of dissolved oxygen around the bottom outlet of the equipment. The elevated oxygen concentration increased the driving force for oxygen transfer to the bottom water. The dissolved oxygen in the bottom water increased from 0.1 mg/L to 2.1 mg/L after the WLAs operated for a week (Figure 8c). However, the high surface water temperature promoted algae growth, so the chlorophyll-*a* concentration in the upper water increased to 9.4 μg/L during the period of single aeration (Figure 8d).

#### 3.4.2. Full Layer Mixing and Oxygenation

To inhibit algal growth and accelerate the destratification processes, the WLAs operated with an air flow rate of approximately 35–40 m^3^/h to achieve the full layer’s mixing and oxygenation starting on 18 September. As Figure 8a shows, the mixing function led to decreased turbidity in the hypolimnion, while increasing the turbidity in the upper water. However, the turbidity in the upper water was still less than 20 NTU, which has little influence on the outflow turbidity. The WLAs delivered the lower water to the surface, leading to a quick decrease in the water temperature in the upper water. The lifted lower water mixed with the upper water and then moved downward because its density remained higher than that of the surface water. This cycle perpetuated the mixing of the upper and lower water layers and led to a gradual increase in the water temperature in the hypolimnion (Figure 8b). Increasing the gas flow rate to the microporous aeration diffusers further increased the dissolved oxygen transfer efficiency, thus leading to a saturated dissolved oxygen concentration around the bottom outlet of the equipment. The disruption of thermal stratification led to a rapid increase of the dissolved oxygen level in the bottom water (Figure 8c). Due to the artificial mixing process caused by the operation of the WLAs, the content of chlorophyll-*a* from the surface to a depth of 10 m consistently decreased, and the algae were brought to the lower-dark layer where they died (Figure 8d). When the WLAs operated until 22 October, the thermal stratification structure was disrupted, and the vertical distribution of chlorophyll-*a* was well-distributed, with the concentration decreasing to 1.5 μg/L.

### 3.5. Good Water Quality Maintained after the WLAs Stopped Running

**Figure 9:**
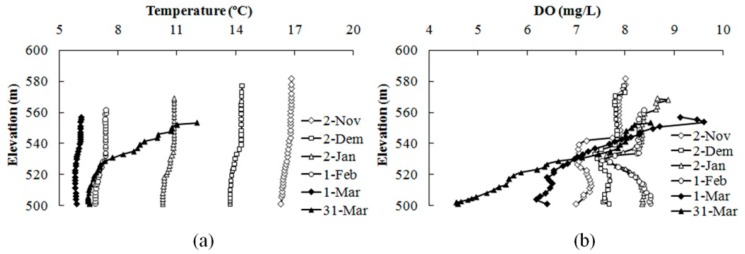
Vertical variation in temperature (**a**) and DO (**b**) in the Heihe Reservoir after the WLAs stopped running.

The reservoir was completely mixed on 18 October, due to the operation of the WLAs, which was three months ahead of the natural mixing time, and the vertical temperature was approximately 17 °C. Then, the WLAs stopped running on 22 October. The surface water temperature decreased with the reduction of the air temperature, leading to an increase in the surface water density. The low-temperature, high-density, and oxygen-rich surface water would continuously be transferred to the lower layer, forming a continuous mixing process in the natural state. The natural mixing process could continue until the surface temperature no longer dropped (estimated at approximately 6 °C) and reached a steady state. The mixing time was advanced, but the temperature at the bottom was increased from 11.67 °C to 17.17 °C, and thus the oxygen consumption rate in the water and the sediment was enhanced. If the mixing oxygen transfer rate was lower than the oxygen consumption rate, the bottom layer would become anoxic again, and the bottom water quality would deteriorate due to the release of pollutants from the sediments. The results show that this assumption was invalid, that the oxygen at the bottom was maintained at a high level after the WLAs stopped running. As Figure 9 shows, the reservoir remained in a mixed state, with a vertical temperature difference smaller than 1 °C and a vertical oxygen concentration larger than 6 mg/L after the WLAs stopped running. With the increase of air temperature, the surface water temperature rose and the vertical temperature difference increased to 5.46 °C on 31 March. The concentration of oxygen at the bottom decreased to 4.5 mg/L due to oxygen consumption by the water and sediment during the thermal stratification process, which indicated the start of a new round of thermal stratification.

## 4. Conclusions

Storm runoff leading to increased dissolved oxygen in the bottom water could effectively inhibit the release of endogenous pollution. However, high inflow volumes carrying a large amount of solids and organic matter increased the oxygen consumption rate in the hypolimnion, and anoxic conditions reappeared after the storm runoff ended and accelerated the release of pollutants. The increased temperature at the bottom induced early mixing in the autumn, which could lead to the deterioration of the whole reservoir water quality due to the strong convective mixing effect.

The operation of the improved water-lifting aerators with an air flow rate of approximately 15–18 m^3^/h could achieve single aeration in the hypolimnion. This function could increase the dissolved oxygen in the hypolimnion while avoiding causing an increase in the turbidity of the upper water. When operating with an air flow of 35–40 m^3^/h, the WLAs achieved the full layer’s mixing and oxygenation functions, which could effectively restrain algal growth and accelerate the destratification processes. Thus, the improved WLAs could ensure water quality during the flood period.

The operation of WLAs led the reservoir to be mixed in advance with an increased vertical mixing temperature. The low temperature, high density and oxygen rich surface water would continuously be transferred to the lower layer, forming a continuous mixing process in the natural state. Thus, good water quality could also be maintained after the WLAs stopped running.

The water quality problem aroused by rainstorms has become severe in the source reservoir, which not only brings risk to the water plants but also seriously affects the city’s water supply safety. The improved water-lifting aerator can not only improve water quality during the operation period but also maintain good water quality after the WLAs stopped running, thus ensure the safety of drinking water for urban residents.
